# Intimate-partner violence and its association with symptoms of depression, perceived health, and quality of life in the Himalayan Mountain Villages of Gilgit Baltistan

**DOI:** 10.1371/journal.pone.0268735

**Published:** 2022-09-21

**Authors:** Gul Nowshad, Neelum Jahan, Nasim Zahid Shah, Nasloon Ali, Tazeen Ali, Sartaj Alam, Ambreen Khan, Mohammad Afzal Mahmood, Malika Saba, Danilo Arnone, Syed M. Shah

**Affiliations:** 1 Center for Clinical Research, University of Massachusetts Medical School, Worcester, MA, United States of America; 2 Aga Khan Health Service, Gilgit, Pakistan; 3 Center of Excellence Women & Child Health, Aga Khan University, Karachi, Pakistan; 4 Institute of Public Health, College of Medicine and Health Sciences, United Arab Emirates University, Al Ain, UAE; 5 School of Nursing and Midwifery/Department of Community Health Sciences, Aga Khan University, Karachi, Pakistan; 6 Harvard Medical School, Harvard University, Boston, MA, United States of America; 7 Department of Behavioral Sciences, Margalla Institute of Health Sciences, Islamabad, Pakistan; 8 School of Public Health, University of Adelaide, Adelaide, Australia; 9 Department of Health, Government of Gilgit Baltistan, Gilgit, Pakistan; 10 Department of Psychiatry and Behaviour Sciences, College of medicine and Health Sciences, UAE University, Al Ain, UAE; 11 Centre for Affective Disorders, Psychological Medicine, Institute of Psychiatry, Psychology and Neuroscience, King’s College London, London, United Kingdom; 12 Department of Family Medicine, Aga Khan University, Karachi, Pakistan; Universidad Pública de Navarra, SPAIN

## Abstract

**Study objectives:**

We aimed to estimate the prevalence of intimate partner violence (IPV) and associated risk factors in married women in rural villages of Gilgit Baltistan in Pakistan.

**Methods:**

A cross-sectional design to assess the magnitude and factors associated with IPV in a random sample of 789 married women aged 18–49 years. A World Health Organization screening instrument was used to assess the presence of IPV in the previous 12 months. A locally validated instrument was adopted to identify self-reported symptoms of major depression according to the DSM IV. Trained nurses obtained socio-demographic and reproductive history through structured interviews. Bivariate and multivariable logistic regression analyses were used to estimate prevalence and identify significant predictors of IPV.

**Results:**

The mean age of the participants was 38.3 years (SD: ±12.8). The prevalence of IPV in women was 22.8% (95% Confidence Interval: 20.0–25.9), 18.5% in pregnant women (95% CI: 11.7–27.9) and significantly associated with depression in 55.1% of IPV cases. Husband education level (college/higher) (Adjusted Odds Ratio: 0.40; 95%CI: 0.22–0.70) and high household income (AOR: 0.44; 95% CI: 0.29–0.68) were protective against IPV. Increase in age (AOR;1.02; 95% CI: 1.01–1.02) and poor relationship with mother-in-law increased the risk of IPV (AOR = 2.85; 95% CI: 1.90–4.28). IPV was positively associated with symptoms of depression (AOR = 1.97; 95% CI:1.39–2.77), poor perceived quality of life (AOR = 3.54; 95% CI: 1.90–6.58) and poor health (AOR = 2.74; 95% CI: 1.92–3.92).

**Conclusion:**

IPV is substantial public health burden significantly associated with depressive symptoms, poor perceived health and the quality of life.

## Introduction

Intimate partner Violence (IPV) against women includes all actions that violate one’s sense of self, physical body, and sense of trust. It involves episodes of physical, psychological (emotional), or sexual violence, perpetrated by a current or former intimate partner [1]. Worldwide, women are particularly vulnerable to this type of violence which is often under-reported [2, 3] and commonly associated with an increased risk of developing physical and mental health conditions, causing significant disability [4]. Psychological consequences amongst women who experience IPV include a range of psychological manifestations which can develop into major depression, suicidal behaviours, anxiety syndromes, post-traumatic stress disorder, disorders of sleep, eating and psychosomatic syndromes [5, 6].

Intimate partner violence also affects children who experience and/or witness violent behaviours at home causing a range of severe and lasting effects [7]. These include learning to normalise gender inequity and attitude towards violence (a prelude to future abusive relationships), becoming victim of educational failure, and a range of psychological problems including substance abuse [8–10].

Prospective follow-up studies have indicated that unplanned pregnancies and having parents with less than a high-school education increase the risk of IPV, while older age and a married status are considered protective [11]. Living in isolated rural areas increases the chance for women to be exposed to IPV [12], whereas access to a wide range of information and communication technologies including radio, computer, fixed or mobile phone, is protective, independent of household wealth, and country socio-economic development [13].

The occurrence of IPV is a significant public health burden in Pakistan [14]. The most tragic and extreme form of IPV towards women takes the shape of ‘honor-related killings’ [15]. In 43% of recorded episodes of ‘honor killing’ the husband was the perpetrator, while a close relative was responsible in the remaining murders [16].

Gilgit-Baltistan (GB) is a highly mountainous region in North Pakistan, with an estimated population of 1,800,000. It is situated amid the mountain ranges of the Himalayas, the Hindu Kush, and the Karakoram, bordering with China, Afghanistan, and India. It consists of seven administrative districts, with each district consisting of several villages. Each village varies in population size ranging from 150 to 500 households.

Women in the remote high mountain villages of GB reported one of the highest suicide rates in Pakistan (33 per 100,00) [17]. Studies have shown that women living in this part of Pakistan are 10 years younger on average than their spouse and have hardly any access to media with only 1.5% in the 15–49 age group reading newspapers, magazines, listening to the radio, or watching television at least once a week [18]. These are known risk factors for IPV which, combined with the high suicide rate, suggest that women in the Himalayan villages of Gilgit Baltistan might be particularly vulnerable. Nevertheless, no study has established the prevalence of IPV and its link with common disorders such as major depression. This is the first study investigating the prevalence of IPV in women who married at least once in their lifetime in the Himalayan villages of GB and the possible relationship with symptoms of depression.

## Methods

### Ethics statement

This study was conducted in collaboration with the Aga Khan Health Service (Neelam Jahan) and the local Government Health Department (Malika Saba). As there is no local ethics committee, the study was done with the permission of the Government Health Department.

Study participants were informed about the study through an information sheet describing the study. All the study participants provided written informed consent. This study was completed as part of the research project named ‘Developed-Developing Countries Partnership for Chronic Non-communicable Diseases (NCDs) Prevention’, and ethics approval for the full project was obtained from the Human Research Ethic Committee of United Arab Emirates University (#CMHS1112).

### Study design & sample size estimation

This study was part of the mental health component of the NCDs prevention project described above. Women aged between 18 and 49 years who married at least once in their lifetime and resided with their husband for at least 12 months in the preceding year were included in the study. Eligible individuals were identified from a list displaying the total number of households in each village of Ghizar district, freely available from the ‘Aga Khan Health Service Pakistan (AKHSP).’ This list was used as sampling frame to recruit the study participants.

The study team included four groups. Each group included a trained nurse, a community health worker, and two volunteers from ‘Silk-Route-To-Healthy Future’, a civil society organization to improve health literacy in the region. A dedicated information sheet describing the research was made available to each study participant when the study was proposed and described. The structured interview questionnaire was administered to the eligible women after obtaining written informed consent. All the interviews were conducted in a place where participants could comfortably and privately answer study questions.

For the purpose of power calculation, the formula for binomial distribution [n = ^z^zα^2^ p (1- p)/d^2^] was adopted. This approach is used in population based cross-sectional surveys, where ‘n’ is the sample size, ‘zα’ is the normal deviate (1.96) at 5% level of significance, assuming a precision or tolerable variation of ±0.03 around the estimated prevalence (P) of IPV in ever married women (13.3%) based on similar studies conducted in Pakistan [19]. The calculation suggested a minimum sample size of 700 participants. The targeted sample size was further increased by a further 25% to 830, to minimise the chance of sampling bias due to non-response.

The sampling frame used a list of villages as enumeration areas. Each village size ranged from 50 to 500 households. By applying a two-stage cluster sampling strategy, one thousand households were selected during the first stage, and the number of the households were selected with a probability proportional to the size of the total number of households in each of the 18 villages. To select households randomly in each of the study villages, we used a modified cluster sampling method [20]. In each study village, a sketch map of the village was prepared with the location of shops, schools, and mosques as village landmarks. A landmark in each village was randomly selected, serving as a starting point. In each of the starting points, a bottle was spun on a hardboard plain surface. The first household in the direction of the open end of the bottle was visited. The next household selected was the one nearest to the first household. This process was repeated until the required number of houses for that village was reached. We used this method in previous studies to estimate cardiovascular risk factors in the study villages [21–24].

### Measures

We used scales already validated in the Pakistani national language (Urdu) to measure the outcomes of interest, including symptoms of depression and intimate partner violence. Furthermore, a standard questionnaire to collect all the relevant information (social and demographic characteristics) was developed and translated in Urdu (with back translation). The questionnaire was carefully designed to include a number of validated questions from our previous studies [21–24] and was pilot tested in a small convenient sample (n = 12) of households in one of the villages to determine its face validity. The interviews were conducted face-to-face in each of the household by a local bilingual research nurse speaking the national language (Urdu) and the local dialect (Shina). A detailed description of the adopted measures is given below.

- ***Sociodemographic characteristics*:** Included age, marital status, number of children, education, monthly household income, employment status, education level of spouse, and any blood relation to the spouse. A historical major events chart was used to further estimate the correct age of the study participants. This is because older adults in the village did not keep birth certificates and could not always recall their age with precision. The degree of consanguinity with the husband was established according to three categories: 1) a first-degree cousin, 2) relative and 3) a non-relative.
- ***Lifestyle variables*:** Included cigarette smoking and other forms of tobacco use. Study participants were classified as smokers if they answered, ‘yes’ to the question *‘*have you ever smoked cigarettes, cigars /biddies/or shisha?’ We also asked questions about the use of ‘Naswar’, prepared from dried tobacco leaves and commonly used as an oral dip.
- ***Self-perceived health***: A self-reported life satisfaction question is routinely used as an indicator of societal well-being in national population-based surveys [25]. Self-perceived health was measured by a single item question and ranging on a 5-point scale from ‘poor’ to ‘fair’, ‘good’, ‘very good’ and ‘excellent’. It is recognised that a perceived health status equivalent to ‘fair’ or ‘poor’ can be associated with chronic medical conditions and/or mental distress [25]. We used this measure in our previous study showing that self perceived poor health was significantly associated with symptoms of depression [21].
- ***Perceived quality of life*:** It was measured by a single question rating the quality of life on a 5-point scale as described above. Studies have shown that this measure can be a meaningful indicator of physical well-being and good mental health [26, 27].
- ***Mother-in-law and daughter-in-law relationship***: The characterization of the ‘mother-in-law /daughter-in-law’ relationship was based on previous wok conducted in Pakistan. [28]. Trained nurses inquired about the ‘mother-in-law’ relationship and measured the response with a a 5-points Likert scale as ‘excellent’, ‘very good, good’, ‘fair’, and ‘poor’.
- ***Measure of intimate partner violence*:** The prevalence of intimate partner violence was defined as physical and/or emotional abuse in the previous twelve months. We assessed intimate partner violence by using an adapted version of the World Health Organization (WHO) domestic violence questionnaire [29]. The adapted version included questions regarding physical and emotional abuse. We excluded sexual abuse related questions due to concerns regarding cultural sensitivity.

Physical abuse was defined as the intentional use of physical force and specific actions such as ‘shaking’, ‘kicking’, ‘slapping with open hands’, ‘beating with fists or with any object’, ‘strangulation’, ‘threats with a knife or gun’, and ‘burning’. Psychological or emotional abuse included questions about ‘yelling’, ‘forced isolation from friends and relatives’, ‘degradation’, ‘insisting on knowing her whereabout at all the time’, ‘threating to harm children or harm someone the woman cares for’, ‘ignoring or treating her with indifference’, ‘suspecting that the woman was unfaithful’, ‘humiliating the woman in front of other people’, ‘saying things that made the woman feel bad about herself’, ‘being angry when the woman spoke to another man’, and ‘inducing fear or emotional harm through words or gesture’.

- ***Measure of depressive symptoms***: Symptoms of depression in the previous two weeks were evaluated using the interviewer-administered Aga Khan University (AKU) anxiety and depression (AKUAD) scale, which is an indigenous depression screening scale, with a ≥20 cut-off score for positive symptomatology [30]. The scale is in the local Urdu language and includes 25 items covering most of the clinical features of depression as specified by DSM-IV criteria, including somatic complaints. At a cut-off score of 20, the scale has 66% sensitivity, 79% specificity, a positive predictive value of 83%, and a negative predictive value of 60% [31]. Responses to the questions were recorded and scored as never (0), sometimes (1), mostly (2), and always (3). The AKUAD scale has been validated using DSM-IV criteria for depression and anxiety as the gold standard criterion for depression and anxiety in Pakistan [32].

### Statistical analyses

Descriptive statistics were used for reporting the prevalence of physical/emotional abuse and included 95% confidence intervals. Chi-square tests were applied to compare differences in proportions of socio-demographic and other predictors of physical and emotional violence. To explore the impact of potential factors on IPV, a binary model was created to estimate intimate partner violence (‘experienced’ or ‘not experienced’ physical/emotional IPV) by using univariate logistic regression as a function of each socioeconomic, demographic, and behavioral factors. Subsequently, multivariable logistic regression was used to determine the independent correlation between predictor variables and physical/emotional IPV. For this purpose, multivariable logistic regression analysis was used to model the dichotomous physical abuse/emotional IPV by the socio demographic and other predictor variables which were statistically significant in bivariate analyses. All statistical tests were two-tailed, and significance level was set at p≤0.05. The results were analysed by using Stata version 13.0 (Stata Corp LP, College Station, TX).

## Results

### Socio-demographic characteristics of the study population

A thousand households were visited until the targeted sample size of 830 potentially eligible homes was reached. Of all the eligible households 789 participant were included, resulting in a response rate of 94.9%. Data collection was completed in 2019.

The average age of participants was 38.3 years (±12.8 SD). The average household nucleus was made of 9 family members with 14.7% having no children, 43.1% ≤ 2 children, and 42.3% ≥ 3 children. The majority of women had no formal education (58.6%), whereas 32.9% obtained secondary level education and 8.5% attended college or higher levels of education. Among the women’s husbands, 31.6% had no formal education, 49.0% received education up to secondary level, and 19.4% achieved college or higher levels of education. Approximately 25.8% of the participants were in consanguineous marriages. Only 7% of the women were employed with an income. Among study participants, 32.2% had a family income in the lowest tertile. The average reported monthly household income was 1200 Pakistani rupees (around US$7.0 or £5.0). Only 5.8% of participants smoked cigarettes, whereas 7.9% used ‘snuff’. The quality of health was reported as good to excellent in 51.0% of cases, whereas quality of life was reported as good to excellent by 94.4% of participants. The relationship with their mother-in-law was described as ‘excellent’, ‘good’, or ‘fair’ by 36.9% of the participants with the remaining 63.1% reporting ‘poor’ or ‘very poor’ quality of the relationship.

### Prevalence of intimate partner violence

Overall, prevalence of IPV was 22.8% (95% CI: 20.0–25.9). A higher proportion of married women reported emotional violence (19.2%; 95% CI: 16.6–22.1) compared to physical violence (9.5%; 95% CI; 7.6–11.7). Participants experiencing IPV had higher prevalence of depression (55.1%; 95% CI:47.6–62.2), compared to non-IPV affected participants (37.3%; 95% CI: 33.5–41.2) (p = 0.00). A significant proportion of pregnant women 18.5% (95% CI: 11.7–27.9) experienced IPV.

### Bivariate and multivariate logistic regression results

Table 1 shows results of the bivariate analysis in relation to the variables of interest as reported by the study participants.

**Table 1:** Prevalence and predictors of intimate partner violence among married women in Himalayan Mountain Villages (n = 789), Pakistan: Bivariate analyses.

Variable		Intimate Partner Violence (physical/emotional)				
		No		Yes		
	**Overall**	**n**	**%**	**n**	**%**	**p value**
**Overall**	789	609	(77.2)	180	(22.8)	
**Age**						
** 18–34**	331	266	(80.4)	65	(19.6)	0.040
** 35–44**	231	181	(78.4)	50	(21.6)	
** ≥45**	227	162	(71.4)	65	(28.6)	
**Education of the participant**						
** No formal schooling**	453	338	(74.6)	115	(25.4)	0.047
** Up to secondary**	254	206	(81.1)	48	(18.9)	
** College or higher**	66	56	(84.8)	10	(15.2)	
**Education of husband**						
** No formal schooling**	247	174	(70.5)	73	(29.5)	<0.001
** Up to secondary**	383	297	(77.6)	86	(22.4)	
** College or higher**	152	132	(86.8)	20	(13.2)	
**Monthly family income**						
** Lowest tertile**	297	214	(72.1)	83	(27.9)	<0.001
** Middle tertile**	234	175	(74.8)	59	(25.2)	
** Highest tertile**	252	215	(85.3)	37	(14.7)	
**Employed**						
** None**	718	556	(77.4)	162	(22.6)	0.709
** Yes**	54	43	(79.6)	11	(20.4)	
**Pregnant**						
** No**	659	505	(76.6)	154	(23.4)	
** Yes**	92	75	(81.5)	17	(18.5)	0.295
**Number of children**						
** None**	115	91	(79.1)	24	(20.9)	0.353
** 1 to 2**	336	265	(78.9)	71	(21.1)	
** ≥3**	330	246	(74.6)	84	(25.4)	
**Blood relation with intimate partner**						
** Closely related 1st or 2nd cousin**	202	162	(80.2)	40	(19.8)	0.026
** Other relative**	250	203	(81.2)	47	(18.8)	
** Nonrelative**	332	241	(72.6)	91	(27.4)	
**Smoke cigarette**						
** No**	743	579	(77.9)	164	(22.1)	0.046
** Yes**	46	30	(65.2)	16	(34.8)	
**Use snuff**						
** No**	723	564	(78.0)	159	(22.0)	0.033
** Yes**	62	41	(66.1)	21	(33.9)	
**Perceived quality of health**						
** Good, very good, or excellent**	401	344	(85.8)	57	(14.2)	<0.001
** Poor, very poor**	385	262	(68.1)	123	(31.9)	
**Perceived quality of life**						
** Good, very good, or excellent**	738	582	(78.9)	156	(21.1)	<0.001
** Poor, very poor**	44	22	(50.0)	22	(50.0)	
**Relation with mother-in-law**						
** Good, very good, or excellent**	284	249	(87.7)	35	(12.3)	<0.001
** Poor, very poor**	486	347	(71.4)	139	(28.6)	
**Positive symptoms of depression AKUAD scale**						
** No**	463	382	(82.5)	81	(17.5)	<0.001
** Yes**	326	227	(69.6)	99	(30.4)	

The prevalence of IPV was significantly higher among participants aged 45 or older (28.6%) and associated with no formal schooling (25.4%), being married to husbands with no formal education (29.5%), living in a household with an income in the lowest tertile (27.9%). These affected women were married to a non-consanguineous husband (27.4%), smoked cigarette (34.8%), or used snuff (33.9%), reported a ‘poor’ or ‘very poor’ relationship with their mother-in-law (28.6%), a self-reported ‘poor’ or ‘very poor’ quality of health (31.9%) and/or life (50%) and self-reported depressive symptoms (30.4%) (all p<0.05).

Multivariate analyses (Table 2) indicated that women exposed to IPV were They were more likely to be older (AOR: 1.02; 95%CI: 1.01–1.02) and to have a poor or very poor relationship with their mother-in-law (AOR = 2.85; 95% CI: 1.90–4.28), a poor quality of health (AOR = 2.74; 95% CI: 1.92–3.92), a poor quality of life (AOR = 3.54; 95%CI: 1.90–6.58), and higher odds of having depressive symptoms (AOR = 1.97; 95%CI:1.39–2.77).

**Table 2:** Intimate partner violence in married women and its correlates in Himalayan Mountain Villages, Pakistan: multivariate logistic regression analyses.

	Intimate Partner violence		
Variable	(Physical/emotional)		
	AOR	95% CI	p value
**Age in years**	1.02	(1.01–1.02)	0.030
**Education of the participant**			
** No formal schooling**	Ref.		
** Up to secondary**	0.75	(0.49–1.14)	0.182
** College or higher**	0.58	(0.28–1.19)	0.140
**Education of husband**			
** No formal schooling**	Ref.		
** Up to secondary**	0.74	(0.51–1.09)	0.130
** College or higher**	0.40	(0.22–0.70)	<0.001
**Monthly family income**			
** Lowest tertile**	Ref.		
** Middle tertile**	0.89	(0.61–1.32)	0.584
** Highest tertile**	0.44	(0.29–0.68)	<0.001
**Pregnancy**			
** Yes**	0.88	(0.49–1.58)	0.680
** No**	Ref.		
**Blood relation with husband**			
** Cousin**	0.92	(0.57–1.47)	0.735
** Relative**	1.49	(0.98–2.29)	0.061
** Not related**	Ref.		
**Relation with mother-in-law**			
** Good, very good or excellent**	Ref.		
** Poor, very poor**	2.85	(1.90–4.28)	<0.001
**Smoke cigarette**			
** No**	Ref.		
** Yes**	1.67	(0.88–3.18)	0.117
**Use snuff**			
** No**	Ref.		
** Yes**	1.57	(0.87–2.81)	0.128
**Quality of health on Likert scale**			
** Good, very good or excellent**	Ref.		
** Poor, very poor**	2.74	(1.92–3.92)	<0.001
**Quality of life on Liker scale**			
** Good, very good or excellent**	Ref.		
** Poor, very poor**	3.54	(1.90–6.58)	<0.001
**Positive symptoms of depression on AKUAD scale**			
** No**	Ref.		
** Yes**	1.97	(1.39–2.77)	<0.001

Conversely, they were less likely to have married a husband with a college level of education or higher (AOR: 0.40; 95%CI: 0.22–0.70) and live in a household with income in the middle or the highest tertile (adjusted OR: 0.44; 95%CI: 0.29–0.68).

## Discussion

We set out to document the prevalence of intimate partner violence (IPV) and its association with symptoms of depression in the remote Himalayan Mountain villages of Pakistan, an area where the rate of suicide in females is believed to be one of the highest in the region. To the best of our knowledge the current study is the first of its kind.

The study shows that the overall ‘past year’ prevalence of IPV experienced by women married at least once in their life was 22.8%. This number was higher than 17.4%, the national average of the ‘past year’ prevalence of physical/emotional prevalence in Pakistan [19]. In the present study the IPV estimates were of a similar magnitude to what reported in global studies in low- and middle-income countries, using a similar criterion of IPV occurrence in the past 12 months [33] including countries such as India (22.5% physical and 11.4% psychological), Nepal (10.0% physical and 7.7% for psychological) and Pakistan (13.6% physical and 20.6% psychological).

Overall, the study population reported a higher prevalence IPV as compared to national estimates.

The study population remained an isolated community with strong traditions and cultural practices until the construction of the China Pakistan Economic Corridor (CPEC), linking this area with mainland Pakistan. The CPEC has contributed to an acceleration of the economic development of the region. However, with economic advantages there has been a gradual disruption of traditional socio-cultural values and family support systems. It is noticeable that the societal transformation has somewhat eroded family cohesion, created higher expectation in many aspects of life including ‘education’, ‘marital matters’, ‘financial aspirations and the increased pressure has negatively affected the mental health of the youngest population resulting in mental health related problems and suicidal behaviours particularly in females [34]. This is consistent with studies conducted globally showing high rates of suicide in ’indigenous populations’, due to the disruption of traditional value systems, cultural norms, and family support systems [35]. The term ‘indigenous’ refers to self contained groups of individuals in specific locations like those living in the remote Himalayan Mountain villages of Pakistan typically characterised by distinct knowledge, language, culture, and ethnic identity [36, 37]. These individuals are particularly at high suicide risk compared to their non-indigenous counterparts according to population-based studies [38].

Our previous work suggests that pregnant women in these Himalayan villages were at higher risk of experiencing depression (48.4%) compared to Canadian Caucasian (8.6%) and Canadian Indigenous (31.2%) pregnant women [21]. The present work suggests that pregnant women are also at risk of experiencing IPV with prevalence figures (18.1%). The notion emerging from our work that one in five women reported experiencing IPV during pregnancy, one of the most critical phases of women’s life, is however concerning and in line with the numbers previously reported by the WHO ‘Multi-Country Study’ on women’s health and domestic violence in Asian countries [29].

In the present study the prevalence of IPV was higher among the participants with no formal schooling (25.4%) versus those with secondary and college or higher level of education (15.2%) and low education was a significant predictor of IPV in bivariate analyses (Table 1). However, after controlling for age and other variables in multivariate analyses, low levels of education in married women were no longer significant. We found independent effects of husband educational attainment on the participant likelihood of reporting IPV. This is in line with previous studies conducted in India and Bangladesh [34, 38].

The study identified that the poorest were at higher risk of IPV, specifically those with a household income in the lowest tertile. The finding highlights the importance of understanding IPV through socioeconomic status. Socioeconomic status can be considered a reflection of other factors such as educational attainment, financial security, and perception of social status. Nevertheless, our findings are in agreement with the WHO ‘Multi-Country Study’ which suggested that high socioeconomic status at household level and secondary level of education are protective against IPV irrespective of country of origin [39]. This study also found that other factors such as alcohol abuse, young age, attitudes supportive of wife beating, having outside sexual partners, experiencing childhood abuse, growing up with domestic violence and other forms of violence during adulthood were positive and statistically significant correlates of IPV in women [40].

The high proportion of children potentially exposed to violence at home because of the number of IPV victims detected in this study might predispose to future IPV. Furthermore, childhood and adolescence are critical times during human development, and it is known that exposure to violence at home bears long-lasting psychological consequences and is related to substance abuse and educational failure [7–10]. Possible ameliorative interventions aiming at IPV reduction in low- and middle-income countries, which include supportive counselling for pregnant women [41], preventative programmes for couples [42] and psychological interventions to address common mental disorders in women experiencing IPV [43] could be particularly useful in this context.

In the present study, women exposed to IPV had almost three times higher odds of having poor or very poor relationship with their mother-in-law (Table 2). In the study population, the family nucleus functions as an ‘extended family system’ and the role of the mother-in-law is critical in controlling family matters, family size, and daily routines including domestic issues. In agreement with previous reports, findings from our work suggest that discord within the ‘extended family system’ increases the risk of violence towards married women [44, 45]. Himalayan societal expectation of the mother-in-law within the ‘extended family system’ is that a married woman must comply and never disagree with the husband and with men in general [46, 47].

The subjugation of women starts early in the high mountain rural community where boys are legitimised to exercise power over their sisters. Studies conducted in other parts of the country have shown that men develop the inappropriate masculine behavior and attitudes towards women [48, 49]. This imposes gender domination in men and hence might provide justification for being violent towards their future wife. This kind of violence brings hopelessness among married women, and 16% of Pakistani women consider suicide to exit the violent environment they find themselves trapped in [33].

Our study clearly demonstrated the wider-ranging negative impact of IPV on women’ affecting physical health, quality of life and psychological well being. The prevalence of depression was almost two-fold higher among participants who experienced IPV as compared to those who did not experience it. This finding is consistent with other studies in rural Bangladesh, showing an increased risk of major depressive episodes among women who experienced IPV [50]. Prospective follow-up studies have shown that IPV is a contributing factor for the development of psychiatric disorders among women [51].

Our study has both limitations and strengths. Given the cross-sectional nature of the work, caution is urged interpreting the relationship between IPV and symptoms of depression. Our data may be subject to recall bias. Disclosure of violence may be inhibited by apprehension of escalating abuse and feelings of embarrassment and shame. Although the interview was conducted by a well-trained nurse in privacy, it is not possible to completely exclude under-reporting. We did not include children aged 15 to 17 years in our study. However, child marriage is not common in the study villages due to high literacy rates among those under twenty years old. We ascertained IPV in the previous 12 months and used caution when comparing results with studies reporting lifetime IPV. The work we present here is complex and this kind of studies are not easy to carry out, especially in the presence of mood disorders [52]. A cross-sectional survey is appropriate to document the prevalence of any given health problem especially when response rate is high (94.9%) compared to other similar population-based studies. The fact that two study co-authors (GN and NJ) have provided trusted routine maternal and child health care in the study area for years is likely to have contributed to the high response rate measured in this work. Birth certificates were not available for all the participants and to avoid bias in the estimation of age we used a local events chart.

## Conclusions

In the present study, the first of its kind in Pakistan, more than one in five married women reported physical and/or psychological violence in the past year. This figure is one of the highest, not just in Pakistan but also worldwide. Women having poor relationships with their mother-in-law, living in low-income households, and married to a husband with a low level of education were more likely to be subject to intimate partner violence. Those exposed to physical/emotional violence were more likely to have poor health and quality of life and more likely to report depressive symptoms. Establishing the prevalence of domestic violence rates in the remote Himalayan Mountain villages of Pakistan is the first essential step to be able to develop effective interventions. Findings of our study suggests the importance of introducing screening mechanisms for women to identify domestic violence. Detecting symptoms of depression might be a way to identify many victims.

A large campaign regarding family relationships is necessary to improve health literacy. This is likely to improve the understanding of concepts related to social equity at an early age and reduce disparities between boys and girls. Access to information might also enhance knowledge of other social realities outside the local community. Focused psycho-social initiatives could contribute to improve couple relationship, address psychological issues in the context of IPV and improve communication within the ‘extended family system’.

Most importantly these Himalayan remote villages would benefit from opportunities to learn about the psychological impact of IPV on the well-being of mothers and children to facilitate recognition and problems resolution. Health care workers could be trained to facilitate dissemination of preventive knowledge, provide early identification of IPV and support affected individuals based on their needs. There are successful examples of health professionals trained to identify early signs of abuse and neglect, resulting in case detection and proficient management [53]. WHO clinical and policy guidelines are readily available to support the creation of a fully aware and trained body of health care workers in any regions of world [53]. The work presented here offers evidence to inform local health care providers in the Himalayan regions of Pakistan to step in and provide the help this vulnerable population needs.
